# Transcriptomic Profile of Mouse Brain Ageing in Early Developmental Stages

**DOI:** 10.3390/brainsci14060581

**Published:** 2024-06-05

**Authors:** Karolina Kulis, Kevin Tabury, Mohammed Abderrafi Benotmane, Joanna Polanska

**Affiliations:** 1 Department of Data Science and Engineering, Silesian University of Technology, 44-100 Gliwice, Poland; karokul256@student.polsl.pl; 2 Radiobiology Unit, Institute for Nuclear Medical Application, Belgian Nuclear Research Centre, 2400 Mol, Belgium; kevin.tabury@sckcen.be (K.T.); rafi.benotmane@sckcen.be (M.A.B.)

**Keywords:** ageing, brain, bioinformatics, neurodevelopment, psychiatric disorders, mus musculus

## Abstract

Ageing is a continuous process that can cause neurodevelopmental changes in the body. Several studies have examined its effects, but few have focused on how time affects biological processes in the early stages of brain development. As studying the changes that occur in the early stages of life is important to prevent age-related neurological and psychiatric disorders, we aim to focus on these changes. The transcriptomic markers of ageing that are common to the analysed brain regions of C57Bl/6J mice were identified after conducting two-way ANOVA tests and effect size analysis on the time courses of gene expression profiles in various mouse brain regions. A total of 16,374 genes (59.9%) significantly changed their expression level, among which 7600 (27.8%) demonstrated tissue-dependent differences only, and 1823 (6.7%) displayed time-dependent and tissue-independent responses. Focusing on genes with at least a large effect size gives the list of potential biomarkers 12,332 (45.1%) and 1670 (6.1%) genes, respectively. There were 305 genes that exhibited similar significant time response trends (independently of the brain region). Samples from an 11-day-old mouse embryo validated the identified early-stage brain ageing markers. The overall functional analysis revealed tRNA and rRNA processing in the mitochondrion and contact activation system (CAS), as well as the kallikrein/kinin system (KKS), together with clotting cascade and defective factor F9 activation being affected by ageing. Most ageing-related pathways were significantly enriched, especially those that are strongly connected to development processes and neurodegenerative diseases.

## 1. Introduction

Ageing is a complex, irreversible process that continues throughout the life of every living being. This process affects many different functions in the body, and several studies have been conducted to address the problem. However, the brain seems to be the most sensitive to time-dependent factors. Therefore, the study of the ageing process, called gerontology, which is the focus of many investigations, is conducted primarily on brain tissue [1,2,3,4]. During the initial stages of neurodevelopment, cellular senescence can be initiated by a number of factors, including DNA damage, oxidative stress, neuroinflammation, and altered proteostasis. These aforementioned processes can impair neurogenesis and neuronal differentiation, potentially contributing to the onset or exacerbation of neurodevelopmental disorders [5,6]. The most commonly observed impacts of ageing are a decline in cognitive and memory functions and the immune system and the onset of neurodegenerative diseases [7]. Experiments reported in the literature are usually conducted on laboratory mice [8], specifically Mus musculus species, which is also the case in this research. It has been confirmed that senescence affects various processes at different times, with some being more resistant to ageing than others. Studies comparing ‘young’ with ‘old’ mice are carried out significantly more frequently. It is, however, often emphasised that further research is needed on the effects of the maturation process on neurodevelopment and behavioural functions, especially in stages before late adulthood (before 12 months old in mice) [9], as this field is still poorly understood and explored. This would help develop approaches that are capable of slowing the harmful effects of ageing and potentially rejuvenating function [10,11]. It has been shown that the first effects of senescence can be observed in mice as early as the fourth month of life [9]. These reported age-related alterations encompass various domains, including auditory and visual perception, synaptic structure, glial modifications, motor learning, and balance. 

We aim to identify brain-ageing-related biomarkers and signalling pathways in a juvenile-to-early-adulthood [9] mouse comparison via advanced statistical and bioinformatics analyses of transcriptomics profiles of mice at different ages (one and six months old), conducted to examine the similarity and dissimilarity of gene expression kinetics across three brain regions (cortex, cerebellum, and hippocampus) and to functionally describe the processes of mouse brain development at the early stage of life. 

## 2. Materials and Methods

### 2.1. Animals

All animal experiments were performed following the European Communities Council Directive (2010/63/EU) and approved by the local ethical SCK CEN (Brainres) or SCK CEN/VITO (ref. 02-012 and 11-005), University of Antwerp and KU Leuven committees. C57Bl/6J were purchased from Janvier (Bio Services, Uden, The Netherlands) or Charles River Breeding Laboratories (Leiden, The Netherlands). Animals were housed under standard laboratory conditions (12 h light/dark cycle), and food and water were available ad libitum. Female mice were coupled during a 2 h period in the morning, at the start of the light phase, to ensure synchronous timing of embryonic development.

### 2.2. Microarray-Based Transcriptomic Profile Measurement

Total RNA was extracted from flash-frozen adult brain regions (cortex, hippocampus, and cerebellum) at 1 or 6 months after birth using the AllPrep DNA/RNA/Protein Mini Kit (Qiagen, Hilden, Germany) and quality-controlled using the 2100 BioAnalyzer (Agilent, Santa Clara, CA, USA). Only samples with an RNA integrity number >8 were hybridised onto Affymetrix Mouse Gene 2.0 ST arrays (Affymetrix, Santa Clara, CA, USA) as per the manufacturer’s recommendations. CEL files were uploaded for subsequent bioinformatics analyses. Data normalisation was performed using a customised Robust Multi-array Average algorithm (background correction for entire probe sequence, quantile normalisation, log2 transformation of intensity signals).

### 2.3. Bioinformatics Analysis

Statistical analysis was performed in Python version 3.10 using different packages for statistics and plotting, including NumPy version 1.26.4, Pandas version 2.2.0, Scikit-learn version 1.3.0, Statsmodels version 0.14.1, and Matplotlib version 3.8.3 or Seaborn version 0.13.2. First, Dixon’s Q test [12] was used to check for outliers. Analysis of variance (ANOVA) was performed to identify the differentially expressed genes. The overall effect size was estimated as eta squared [13], defined as the proportion of the total variation in the outcome variable that can be attributed to the chosen factor. Two data analysis scenarios were applied: (1) multiple one-way time-related ANOVA analyses were performed to derive genes called the ageing markers that are specific to each brain region separately, and (2) two-way ANOVA, with tissue region and time as the independent variables and tissue region: time as the interaction components, to identify tissue- and time-dependent-only biomarkers (defined as those with no significant other factor and interaction components). The trend over time was examined for genes identified as time-dependent-only. The Venn diagrams summarise the analyses. Functional analyses using Over-Representation Analysis (ORA) and Gene Set Enrichment Analysis (GSEA) [14,15] were performed to identify age-enriched signalling pathways. It was mainly carried out using the R programming language version 4.3.1 and the fgsea package version 1.26.0 provided by Bioconductor. The Reactome [16] and STRING [17,18] services were also utilised. The Molecular Signatures Database (MSigDB) was used to collect the gene sets related to senescence, brain development, and psychiatric disorders. All genes identified as potential ageing markers were ranked according to ANOVA’s effect *p*-value or the effect size value of the time factor. 

### 2.4. Data Description

The gene expression levels of 27,352 transcripts, assessed in different brain regions (cortex, hippocampus, and cerebellum) and collected at various time points (1 and 6 months after birth) in C57bl mice, constituted the data. There were 18 samples available (each experiment performed for a tissue–time pair was performed on three mice). However, the study of this project was conducted on 17 samples, of which one tested positive as an outlier and was therefore removed (see Supplementary Figure S1). In addition, three samples from mice on embryonic day E11 were incorporated in the final stages of analysis to validate the obtained results.

## 3. Results

The two-way ANOVA analysis revealed a stronger effect of brain tissue region over the natural ageing process. A total of 16,374 genes (59.9%) significantly changed their expression levels, among which 7600 (27.8%) demonstrated tissue-dependent and time-independent differences, and 1823 (6.7%) demonstrated time-dependent and tissue-independent responses classified as potential biomarkers of ageing. The exemplary over-represented signalling pathways in the time-dependent-only biomarkers are tRNA and rRNA processing in the mitochondrion [19,20] and contact activation system (CAS) [21], as well as the kallikrein/kinin system (KKS) [22], together with clotting cascade and defective factor F9 activation [23] (see Supplementary Figure S2, Table S1). 

While each brain region was analysed separately, slightly weaker changes in time were observed for the cerebellum (3347 genes), with the cortex and hippocampus being at almost similar levels (3694 and 4007, respectively). Among the signalling pathways, the most affected were the noncanonical activation of NOTCH3 [24] (for the hippocampus) and the intrinsic (mitochondria-dependent) pathway of apoptosis [25] (for the cerebellum). Only 305 genes demonstrated a similar trend in time of significant changes across all brain tissue regions. These were linked to the chromatin organisation pathways (HAT acetylate histones) [26], metabolism of proteins (neddylation) [27], amyloid fibre formation [28], and association of TriC/CCT with target proteins in biosynthesis [29]. The deregulation of the brain-ageing-related process of the YAP1- and WWTR1 (TAZ)-stimulated gene expression loop [30] was also observed. Additionally, we noticed the activation and oligomerisation of the Bak protein [31], as well as the over-representation of genes involved in the TBC/RABGAPs activation pathway [32] (see Supplementary Figure S3, Table S2). 

**Figure 1:**
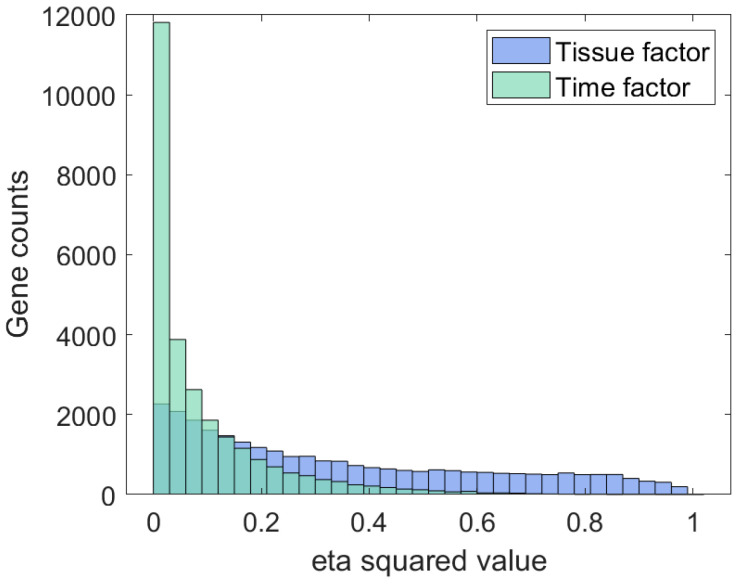
Distribution of effect size measures for tissue and time factor.

A two-way ANOVA analysis, modelling the main effects and interaction components, was performed to adjust for the observed dependency of the tissue region and time response. Moreover, post hoc testing was applied to reveal endpoint differences. Since the integrative analysis of two-way and one-way ANOVAs causes problems because of the different statistical powers of those tests in the case of small sample sizes, the effect size analysis was performed in parallel. Based on the eta-squared (η^2^) effect size measures obtained per gene and both main factors (tissue region and time), the histograms were constructed and are presented in Figure 1.

**Figure 2:**
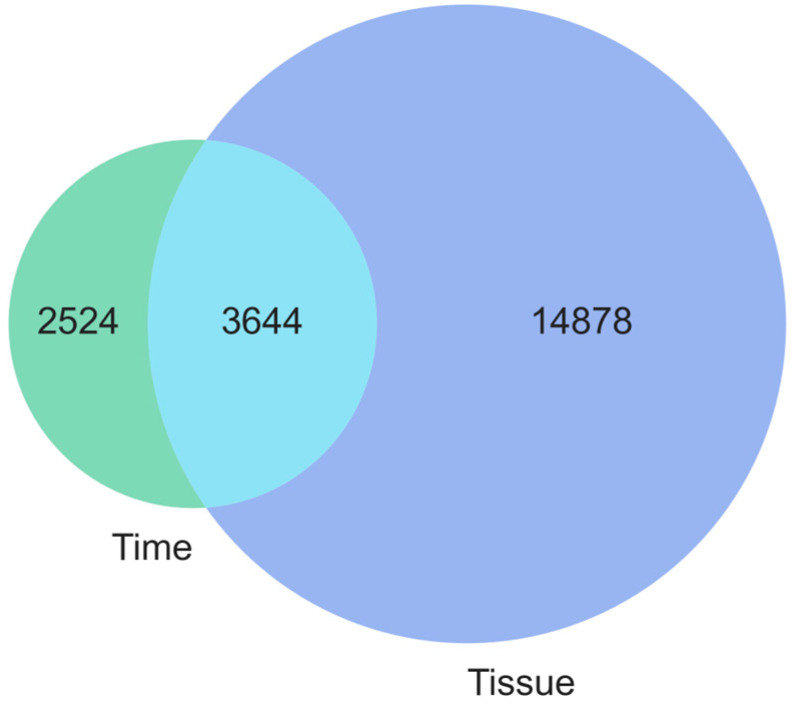
Venn diagram presenting how many genes had at least a large effect size for the time and tissue factors.

One can notice that for the tissue factor (coloured blue), the distribution is significantly more skewed to the right compared to the time-related one, with more genes with at least a large effect size (η^2^ > 0.14). The similarity of the obtained gene sets is summarised in the Venn diagram in Figure 2.

**Figure 3:**
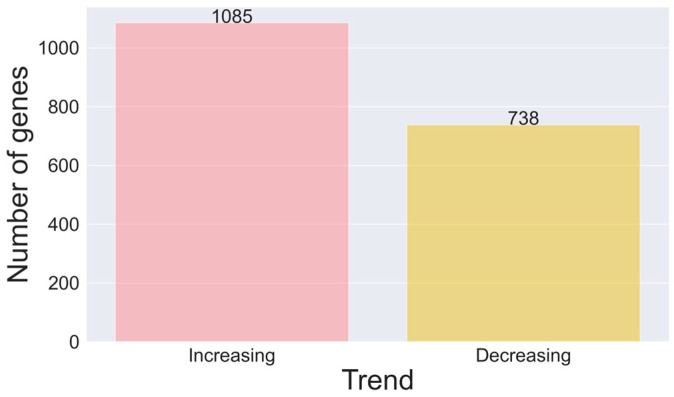
Barplot presenting the gene trend, selected as tissue-independent ageing markers.

To identify potential brain ageing biomarkers, we required genes to show statistically significant time dependence only (no significant dependence on the tissue region and no significant interaction component between the tissue region and time), which resulted in 1823 such genes. Each marker’s average gene expression level was compared between one-month-old and six-month-old mice to determine the trend. In most cases (1481 genes, 64.5%), the gene expression increased over time (see Figure 3). The protein–protein interaction network was recognised by the STRING service (see Supplementary Figure S4). The network effectively clusters the genes, indicating functional relationships or connections between the proteins represented in the data. The PPI Enrichment *p*-value for this network is <1 × 10^−16^.

As was mentioned above, GSEA (Gene Set Enrichment Analysis), a powerful computational method, provides a high-level view of biological pathways and illuminates their importance in different processes. This analysis can reveal complex molecular signatures and patterns reflecting fundamental molecular processes. 

**Figure 4:**
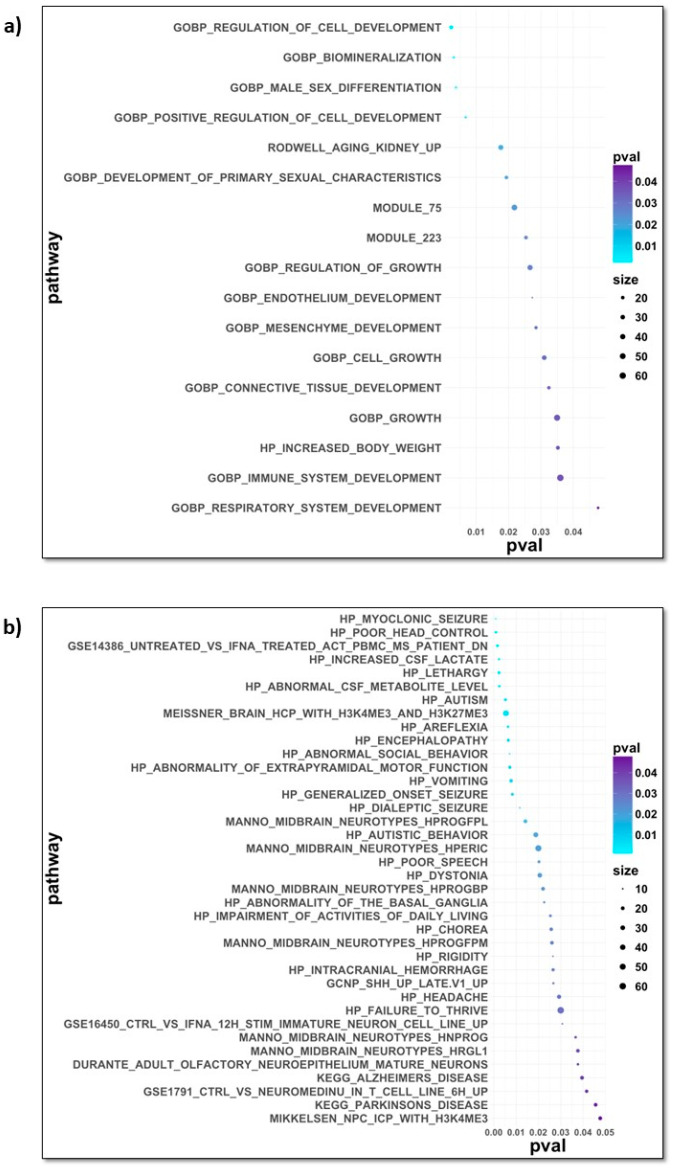
Dot plots with pathways of GSEA revealing a statistically significant connection with the factors of (**a**) ageing and (**b**) neurodevelopment and psychiatric disorders.

For the identified set of 1823 potential ageing markers, we focused on analysing the pathways related to ageing and neurodevelopment and any psychiatric diseases resulting from various neurological causes only. Many of these pathways showed statistical significance, meaning that senescence significantly impacts neurological changes. The results can be studied in more detail on the dot plots in Figure 4 and in the Supplementary Tables S3 and S4 (for ageing and chosen diseases, respectively). Some of the age-related neurodegenerative disorders that were found to be enriched, such as Alzheimer’s or Parkinson’s disease, are consistent with what has already been reported in the literature [7,33].

**Figure 5:**
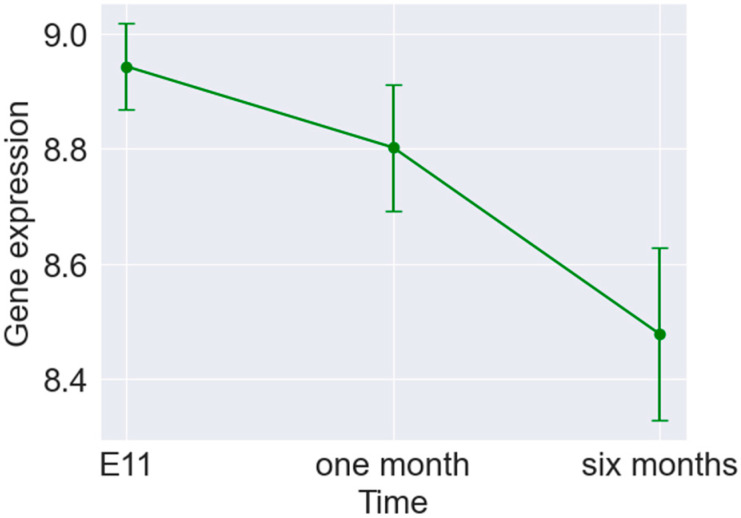
Error bar plot presenting the changes in the mean gene expression over time for one of the identified markers of ageing (Syngr2).

This study aims to understand the transcriptomic changes during early developmental stages. To further expand on this matter and validate the selected set of ageing biomarkers, samples from the embryos were included in the analysis. The selected ageing biomarkers remained time-dependent with increasing/decreasing trends and large effect sizes. This confirms that the identified genes can be considered markers of ageing, influencing organisms at the early stages of life. A plot displaying the changes in mean gene expression over time for one of the identified markers of ageing (Syngr2) is presented in Figure 5, along with corresponding error bars indicating the standard deviations.

## 4. Discussion

When investigating the topic of ageing and its effects on the body’s processes, studies often compare young mice with old mice [8]. In contrast, there are fewer reports on the effects during the early stages of life. This issue has been addressed in the literature [9]. This study aims to examine the changes between the first and sixth months of a mouse’s life, classifying them as juvenile and adult, to better understand the effects of ageing. Therefore, this research focuses on a less studied area, examining the transformations that occur on a specific timescale. Understanding these changes may help develop approaches to slow the adverse effects of ageing. Previous research has shown that senescence-related effects can occur in mice as early as four months of age [9]. This research suggests that specific dysfunctions related to neurodevelopment may hurt early developmental stages, potentially leading to significant psychiatric changes.

In the investigation described in this study, a series of analytical procedures were employed to identify genes that may be referred to as potential markers of ageing. Subsequently, these identified genes were compared with those that have previously been documented in the literature to ascertain their concordance with genes that are known to be influenced by senescence [2,34]. Some of the common ones involve Syngr2 (Synaptogyrin 2; in neuronal cells, modulates the localisation of synaptophysin), C4a (Complement C4A (Chido/ Rodgers Blood Group); related to Regulation of Insulin-like Growth Factor (IGF) transport and uptake by Insulin-like Growth Factor Binding Proteins (IGFBPs)), Ifitm3 (Interferon-Induced Transmembrane Protein 3; among its related pathways are Cytokine Signalling in Immune system and Antiviral mechanism by IFN-stimulated genes), Rtp4 (Receptor Transporter Protein 4; predicted to enable olfactory receptor binding activity), Bst2 (Bone Marrow Stromal Cell Antigen 2; its target viruses belong to diverse families, including retroviridae, flavivirideae, arenaviridae, rhabdoviridae, and orthomyxoviridae), or Gbp3 (Guanylate-Binding Protein 3; Interferon (IFN)-inducible GTPase that plays important roles in innate immunity against a diverse range of bacterial, viral, and protozoan pathogens).

There has been some evidence [1,35] that the immune status of the meninges may be affected by the ageing process [36], which in turn may impact T lymphocytes and B cells. In a further investigation, the genes identified in this article as markers of ageing were subjected to further study [34] to identify their effect on this area. As a result, it was found that several genes from this study were strongly associated with the regulation of T lymphocytes and B cells and, therefore, have a significant impact on the immune system’s function. The identified ageing markers include the following genes: Runx3 (RUNX Family Transcription Factor 3; regulates T cell proliferation and differentiation), Tfrc (Transferrin Receptor; required for neurologic development; positively regulates T and B cell proliferation), Psmb11 (Proteasome Subunit Beta 11; critical for CD8-positive T cell development), and Ly75 (Lymphocyte Antigen 75; causes reduced proliferation of B-lymphocytes).

Some of the age-related neurodegenerative disorders that were found to be enriched, such as Alzheimer’s or Parkinson’s disease, are consistent with what has already been reported in the literature [7,33]. As demonstrated, changes leading to neurodegenerative diseases can be observed in mice as early as six months of age. Consequently, this may suggest that these processes begin already in adolescence. Nevertheless, they are slow to develop, so the diseases do not manifest until later.

Many genes identified in this study as ageing markers are associated with psychiatric and neurodevelopmental disorders. Some examples include [34] Stub1 (STIP1 Homology And U-Box-Containing Protein 1; mutations in this gene cause spinocerebellar ataxia), Pdgfrb (Platelet-Derived Growth Factor Receptor Beta; diseases with it associated include Kosaki Overgrowth Syndrome and Premature Aging Syndrome, Penttinen Type; present in the HPO’s CHOREA, DYSTONIA, and RIGIDITY gene sets), Ndufa10 (NADH: Ubiquinone Oxidoreductase Subunit A10; diseases associated with this gene include Leigh Syndrome, among others), Katnb1 (Katanin Regulatory Subunit B1; diseases associated with KATNB1 include Lissencephaly 6 With Microcephaly and Lissencephaly 2), Lias (Lipoic Acid Synthetase; diseases associated with this gene include Hyperglycinemia, Lactic Acidosis, And Seizures and Lipoic Acid Synthetase Deficiency), and Ptpa (Protein Phosphatase 2 Phosphatase Activator; diseases associated with PTPA include Parkinson’s Disease 25, Autosomal Recessive Early-Onset, With Impaired Intellectual Development and Tauopathy).

The main over-represented signalling pathways in the common ageing biomarkers that were mentioned at the beginning of the Results section were examined to determine whether they are related to the topic of ageing in the literature. Disruptions in mitochondrial tRNA and rRNA processing, which are crucial for maintaining mitochondrial function, have been linked to brain ageing and neurodegenerative diseases. This highlights the potential of these processes as therapeutic targets for extending the brain’s lifespan [19]. Moreover, the kallikrein–kinin system (KKS) has been associated with the pathogenesis of neurological disorders due to its involvement in mediating several pathophysiological features and regulating diverse aspects of brain function. Experiments have demonstrated that modulation of KKS receptors (B1R and B2R) may result in deleterious pathological effects in the context of neurological disorders [22]. Furthermore, studies have identified platelet factors as potential therapeutic targets for reducing inflammation and improving cognitive function in the elderly [37].

The study’s main limitations are the relatively small sample size and the lack of an external validation dataset. We addressed these issues by supporting statistical testing with the effect size analysis and by including the embryonic brain gene expression profile in the validation stage. 

## 5. Conclusions

This work examined the effects of time in the early life stages of an organism, focusing on neurological and developmental functions. The aim was to identify potential genes that could serve as markers of the ageing process. The analyses conducted confirmed the impact of time on the brain’s transcriptomic profile. The study identified several time-related biological pathways associated with the organism’s development in the early stages, as well as functions related to neurodevelopment and neurodegenerative and psychiatric disorders. Including embryonic mouse samples in the study yielded significant effect sizes, which validates the use of ageing markers.

## Data Availability

No new data were created or analyzed in this study. Data sharing is not applicable to this article.

## Supplementary Materials

The following supporting information can be downloaded at https://www.mdpi.com/article/10.3390/brainsci14060581/s1: Figure S1: The 2D UMAP projection of all collected samples. Figure S2: The exemplary overrepresented REACTOME signalling pathways in the time-dependent only biomarkers (two-way ANOVA). Figure S3: The exemplary overrepresented REACTOME signalling pathways in different brain tissue-regions (one-way ANOVAs). Figure S4: The protein–protein interaction network; Table S1: GSEA results with significant pathways related to ageing (the time-dependent only biomarkers, two-way ANOVA); Table S2: GSEA results with the overrepresented signalling pathways in different brain tissue-regions (one-way ANOVAs). Table S3: List of significantly GSEA overrepresentaed significant pathways related to ageing. Table S4: List of significantly overrepresented signaling pathways related to neurodevelopment and psychiatric disorders.
