# Supplementary figures and images for: Transcriptomic Profile of Mouse Brain Ageing in Early Developmental Stages
Source: Brain Sci. 2024 Jun 5;14(6):581. doi: 10.3390/brainsci14060581 (PMC11201909; doi:10.3390/brainsci14060581)

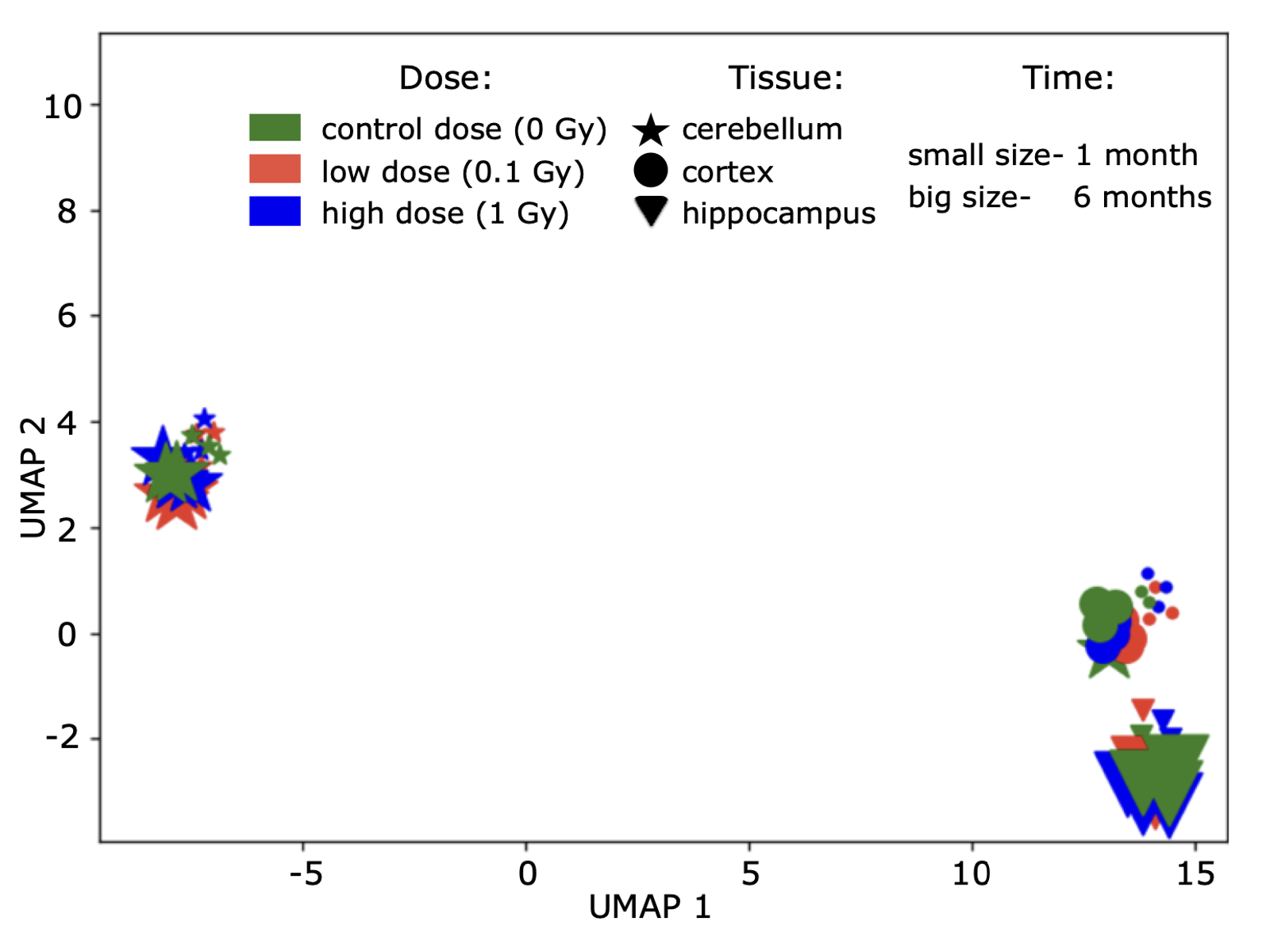

Supplement: Supplementary file 1 [file brainsci-14-00581-s001.zip › Figure_S1.png]

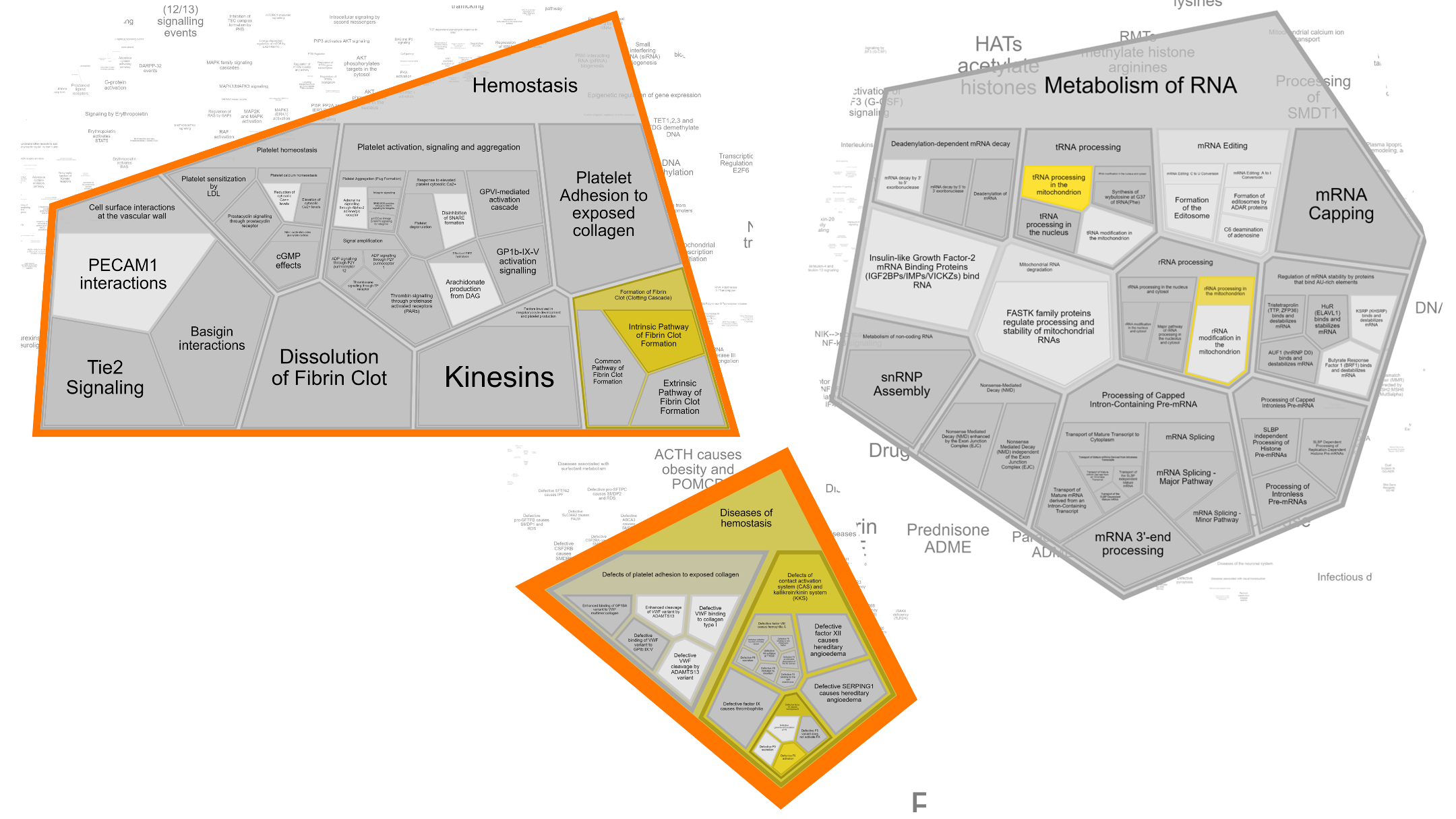

Supplement: Supplementary file 1 [file brainsci-14-00581-s001.zip › Figure_S2.png]

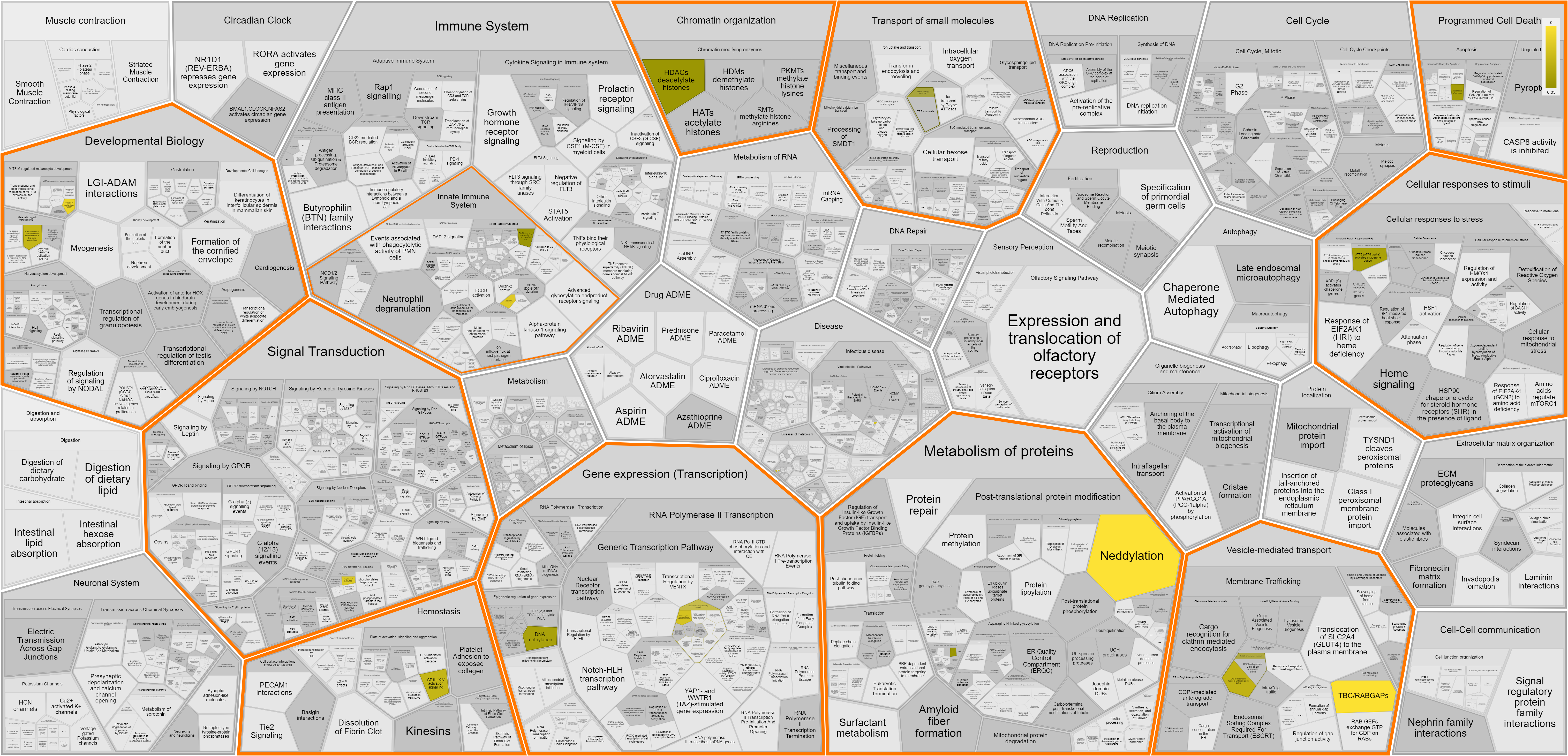

Supplement: Supplementary file 1 [file brainsci-14-00581-s001.zip › Figure_S3.jpg]

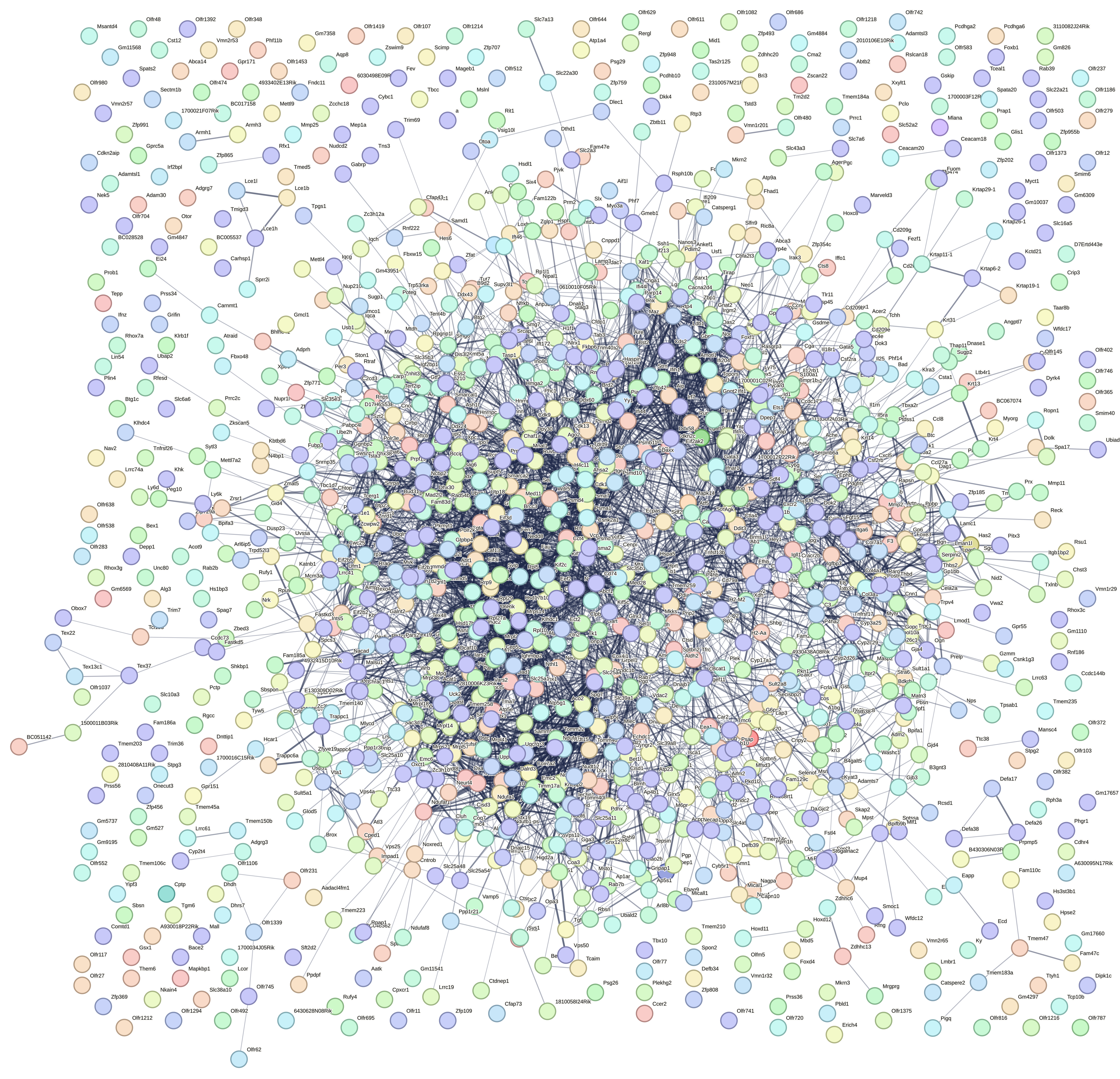

Supplement: Supplementary file 1 [file brainsci-14-00581-s001.zip › Figure_S4.png]
